# Examination of the Feasibility, Acceptability, and Efficacy of the Online Personalised Training in Memory Strategies for Everyday Program for Older Adults: Single-Arm Pre-Post Trial

**DOI:** 10.2196/41712

**Published:** 2023-04-20

**Authors:** Kerryn Pike, Carl I Moller, Christina Bryant, Maree Farrow, Duy P Dao, Kathryn A Ellis

**Affiliations:** 1 School of Psychology and Public Health La Trobe University Melbourne Australia; 2 John Richards Centre for Rural Ageing Research La Trobe University Wodonga Australia; 3 School of Applied Psychology Griffith Centre for Mental Health & Menzies Health Institute Queensland Griffith University Gold Coast Australia; 4 Melbourne School of Psychological Sciences The University of Melbourne Melbourne Australia; 5 Wicking Dementia Research and Education Centre University of Tasmania Hobart Australia; 6 Academic Unit for Psychiatry of Old Age Department of Psychiatry The University of Melbourne Melbourne Australia

**Keywords:** cognition, learning, internet-based intervention, social support, subjective cognitive decline, mobile phone

## Abstract

**Background:**

Memory strategy training for older adults helps maintain and improve cognitive health but is traditionally offered face-to-face, which is resource intensive, limits accessibility, and is challenging during a pandemic. Web-based interventions, such as the Online Personalised Training in Memory Strategies for Everyday (OPTIMiSE) program, may overcome such barriers.

**Objective:**

We report on OPTIMiSE’s feasibility, acceptability, and efficacy.

**Methods:**

Australians aged ≥60 years reporting subjective cognitive decline participated in this single-arm pre-post web-based intervention. OPTIMiSE is a 6-module web-based program offered over 8-weeks with a 3-month booster. It has a problem-solving approach to memory issues, focusing on psychoeducation about memory and aging, knowledge and practice of compensatory memory strategies, and personalized content related to individual priorities. We examined the feasibility (recruitment, attrition, and data collection), acceptability (recommendation to others, suggestions for improvement, and withdrawal reasons), and efficacy (change in goal satisfaction, strategy knowledge and use, self-reported memory, memory satisfaction and knowledge, and mood; thematic content analysis of the most significant change; and the application of knowledge and strategies in daily life) of OPTIMiSE.

**Results:**

OPTIMiSE was feasible, demonstrated by strong interest (633 individuals screened), a satisfactory level of attrition (158/312, 50.6%), and minimal missing data from those completing the intervention. It was acceptable, with 97.4% (150/154) of participants agreeing they would recommend OPTIMiSE, the main suggestion for improvement being more time to complete modules, and withdrawal reasons similar to those in in-person interventions. OPTIMiSE was also efficacious, with linear mixed-effects analyses revealing improvements, of moderate to large effect sizes, across all primary outcomes (all *P*<.001): memory goal satisfaction (Cohen *d* after course=1.24; Cohen *d* at 3-month booster=1.64), strategy knowledge (Cohen *d* after course=0.67; Cohen *d* at 3-month booster=0.72) and use (Cohen *d* after course=0.79; Cohen *d* at 3-month booster=0.90), self-reported memory (Cohen *d* after course=0.80; Cohen *d* at 3-month booster=0.83), memory satisfaction (Cohen *d* after course=1.25; Cohen *d* at 3-month booster=1.29) and knowledge (Cohen *d* after course=0.96; Cohen *d* at 3-month booster=0.26), and mood (Cohen *d* after course=−0.35; nonsignificant Cohen *d* at booster). Furthermore, the most significant changes reported by participants (*strategy use, improvements in daily life, reduced concern about memory*, *confidence and self-efficacy*, and *sharing and shame busting with others*) reflected the course objectives and were consistent with themes arising from previous in-person interventions. At the 3-month booster, many participants reported continued implementation of knowledge and strategies in their daily lives.

**Conclusions:**

This feasible, acceptable, and efficacious web-based program has the potential to enable access to evidence-based memory interventions for older adults worldwide. Notably, the changes in knowledge, beliefs, and strategy use continued beyond the initial program. This is particularly important for supporting the growing number of older adults living with cognitive concerns.

**Trial Registration:**

Australian New Zealand Clinical Trials Registry ACTRN12620000979954; https://tinyurl.com/34cdantv

**International Registered Report Identifier (IRRID):**

RR2-10.3233/ADR-200251

## Introduction

### Background

Many older adults report changes in their cognition, particularly their memory [1-3]. These changes can elicit negative emotional responses (anger, embarrassment, and frustration) and impact the person’s sense of self; relationships and social interactions; and engagement in valued personal, work, and leisure activities [4,5]. Subjective cognitive concerns have also been linked to an increased risk of future cognitive decline and dementia [6,7]. Thus, understandably, cognitive concerns in older adults have been linked to reduced quality of life [8,9].

Several systematic reviews and meta-analyses have provided good evidence that memory interventions can alter how older adults feel about their cognition, improve their confidence and self-efficacy, impact their relationships, and improve their well-being and quality of life [10-13]. A core component of effective memory interventions for older adults is the facilitation of the normalization of age-related cognitive changes [14]. This shift in how older adults perceive their cognition can be achieved through psychoeducation regarding how memory works and normal aging processes, and by providing opportunities for comparison with peers [15]. Psychoeducational groups are considered an ideal setting for facilitating normalization, as they provide an opportunity to interact with others similar to oneself [14], which has been shown to improve well-being [15].

Another core component of effective interventions is practical skill building, that is, training older adults in the use of effective memory strategies. This incorporates a focus on compensatory strategies to manage memory difficulties in everyday life rather than a restorative approach involving repeated practice on a discrete cognitive task to improve performance in that domain [16]. To enhance the translation of this training to everyday life, it is important to focus on training in ecologically relevant tasks that older adults are motivated to improve, such as remembering names or prospective memory [17]. Effectiveness can be enhanced by embedding behavior change techniques, such as explaining the reasoning behind the use of the skills (ie, psychoeducation around memory models); demonstrating the skill; supporting participants to practice the skill, both in session and at home; and supporting participants to solve any issues that arise [18].

Although the benefits of these memory interventions are well established, most of the studies to date have been based on in-person, face-to-face group models. Although this may not be an issue for a research study, it can cause difficulties in terms of feasibility and sustainability when the interventions are implemented in a wider clinical or community setting. These interventions tend to be resource intensive in terms of staffing, facilitator training, and administrative demands [19]. Staff availability and turnover can further reduce the sustainability of the interventions [19]. In addition, face-to-face groups create access barriers, as participants need to be available at specific times and dates, often for many weeks, and be able to attend a particular location [20]. This can be a particular challenge for older adults, who are more likely to have chronic health conditions and reduced mobility. It is also an issue for those with limited transport options or those residing in rural and remote regions.

These access and resource issues can potentially be addressed by delivering an intervention in a web-based format [20]. A web-based format for memory interventions appears feasible for older adults [20]; indeed, it appears to have become even more feasible owing to the increased familiarity and comfort with using technology during social distancing because of the COVID-19 pandemic [21]. Furthermore, a recent survey demonstrated that web-based memory strategy training was the web-based intervention of most interest to older adults, with 82% of the respondents expressing a strong interest in this type of content [22]. There are many examples of older adults using computerized programs for cognitive interventions, both in a laboratory setting or on the web [23,24]. Most of these programs follow a restorative approach involving repeated practice of a task to improve performance, which has little evidence for generalization to everyday life [25]. A recent example of a mixed restorative and compensatory approach compared a web-based program to a classroom-based equivalent and found no differences in efficacy or satisfaction with the training [26]. Of note, for both the web-based and classroom interventions, the authors reported no effect on the transfer of training effects to the “real world” nontrained cognitive abilities and no improvement in memory or everyday function compared with a control group. Another recent example focused on psychoeducation and a compensatory strategy approach, adapting a facilitator-guided, in-person memory intervention for older adults into a self-guided e-learning program [27]. The authors found that this intervention was feasible (68% completion rate) and acceptable (high levels of participant-reported satisfaction) for healthy older adults, 64% of whom were worried about declining memory. Pre-post intervention measures indicated improved goal satisfaction and decreased concern about memory changes. After the intervention, most participants also reported increased confidence in memory, use of memory strategies, and health-promoting lifestyle changes. The long-term maintenance of the changes was not assessed.

### Goal of This Study

Given that the overall aim of memory interventions is to improve function in everyday life, we aimed to develop a new web-based memory intervention incorporating key elements of interventions that are effective in shifting how older adults feel about their memory, leading to increased confidence, mood, and quality of life. Thus, we created the Online Personalised Training in Memory Strategies for Everyday (OPTIMiSE) program [16]. The primary aim of this pilot trial was to evaluate the feasibility, acceptability, and efficacy of the OPTIMiSE intervention for older adults with cognitive concerns. We aimed to determine whether our new web-based program could potentially enable greater access to memory interventions for older adults with cognitive concerns. In terms of efficacy, we were interested in changes occurring immediately after course completion in goal satisfaction, strategy knowledge and use, self-reported memory, memory satisfaction and knowledge, and mood, as well as the most significant change reported by participants. We were also interested in whether changes in these outcome measures were maintained 3 months later and whether participants had been able to apply the knowledge and strategies from OPTIMiSE in everyday life.

## Methods

### Overview

A protocol paper containing the full design and methods of this study has been previously published [16]. This paper is focused on reporting the primary aim of the trial, as well as the mood symptom outcomes. Data addressing the secondary aims will be reported in a future manuscript. The key details of the method are summarized below.

### Study Design

This pilot trial was a single-arm pre-post study [28], followed by a single maintenance session 3 months after the intervention. It was registered with the Australian New Zealand Clinical Trials Registry (ACTRN12620000979954). Reporting follows the Strengthening the Reporting of Observational Studies in Epidemiology reporting guideline for cohort studies [29] and the CONSORT (Consolidated Standards of Reporting Trials) extension for pilot and feasibility trials [30], with adaptation of items where necessary to reflect the study’s nonrandomized design.

### Ethics Approval

The study was approved by the relevant institutional Human Research Ethics Committees (La Trobe University HEC 20025 and University of Tasmania Ethics Committee 20323). All the participants provided informed consent before completing any of the outcome measures. All the published study data were deidentified. No compensation was offered for participating in the study.

### Participants

Participants were recruited from around Australia through emails to University of the Third Age (U3A) groups, Probus clubs, and individuals who had previously expressed interest in attending a face-to-face La Trobe and Caulfield Hospital (LaTCH) memory strategy program [31] but were unable to attend. The following inclusion criteria were applied: individuals (1) aged ≥60 years; (2) reporting subjective cognitive decline (responding yes to the question, “Do you think your memory or thinking is worse than it was 10 years ago?”); (3) sufficiently fluent in reading and typing English to access, read, and comprehend the course material and participate in text-based, web-based discussions; (4) able to complete OPTIMiSE during the set 8-week period; and (5) able to complete the web-based evaluation questionnaires without assistance. Participation was limited to the current residents of Australia. Participants were excluded if they self-reported any of the following: (1) diagnosis of dementia, (2) diagnosis of a current psychiatric disorder likely to impact cognition (eg, psychotic illness or severe depression), (3) history of any neurological condition likely to impact cognition or study participation (eg, stroke, cerebral palsy, epilepsy, multiple sclerosis, Parkinson disease, and moderate or severe traumatic brain injury), and (4) current alcohol or drug dependency.

Our recruitment goal was informed by a power analysis that suggested that 107 participants would need to complete preintervention and postintervention testing, given an expected effect size from a meta-analysis of memory training [32] in older adults of Hedges *g*=0.243, with power=0.80 and α=.05. Allowing for an attrition rate of 30% [33], we, therefore, aimed to recruit at least 153 participants for this pilot study.

### Intervention

OPTIMiSE was a free 6-module web-based course (plus an introductory and conclusion module) of approximately 2 hours of content per week, delivered through the Massive Open Online Course platform at the University of Tasmania Wicking Dementia Centre. It ran over an 8-week period (from October 20, 2020, to December 15, 2020), with a new module released weekly over the first 6 weeks. Participants could complete the modules at any time within the 8-week period. There was a booster session 3 months after the completion of the course (open between March 16, 2021, and March 30, 2021). The intended learning outcomes of OPTIMiSE were for the participants to (1) understand memory, how it works, and how it changes across the life span and (2) learn and apply effective memory strategies for everyday life.

OPTIMiSE was developed by the core OPTIMiSE team in consultation with a stakeholder advisory committee comprising consumers, service providers, and clinician-researchers. Core program content was adapted from key elements of successful memory strategy interventions, such as the LaTCH program [31], that are informed by the refined Theoretical Domains Framework [34]. OPTIMiSE provided psychoeducation about how memory works, reasons for forgetting, normal aging, well-being in later life, and sleep. OPTIMiSE focused on compensatory strategies rather than a restorative approach and provided specific skill training in strategies for improving memory and well-being. Skill training targeted everyday tasks that people are generally motivated to improve, with a problem-solving approach to understanding why a difficulty was occurring and the use of this understanding to select the best strategy to manage that issue.

Content was provided using a conversational framework through videos (including interviews with guest experts), text, static and interactive diagrams, LEGO (The LEGO Group) animated case studies, photographs, summaries, transcripts, reflective notes, quizzes to check learning, and links to external resources (websites, videos, articles, and help sheets). Participants were provided with worked examples of how to use core strategies, followed by web-based examples to try themselves. Each module ended with homework, which participants were asked to complete and then discuss on the moderated discussion boards—either strategies for participants to try in their daily lives or an opportunity to reflect on how the provided information applied to them. The discussion boards also aimed to provide participants with an opportunity to share their experiences of memory changes with their peers. As outlined in our protocol paper [16], personalized experience was provided through optional content and customized homework activities based on each participant’s nominated memory priority.

### Outcome Measures

#### Feasibility

The feasibility of the intervention was determined by recruitment rates (number of interested participants, number of organizations contacted, time frame for recruitment, number of participants who gave informed consent out of the participants who expressed interest), attrition (percentage of recruited participants who completed OPTIMiSE), and data collection (amount and nature of missing data).

#### Acceptability

The primary measure of acceptability was whether participants would recommend OPTIMiSE to others (measured as the percentage of participants who selected agree or strongly agree to this question on the postcourse evaluation). Acceptability was also assessed by reviewing participants’ suggestions for improvements, which were gathered as part of the postcourse evaluation survey. In addition, all participants who elected to withdraw from the study were invited to complete a short web-based survey regarding their reasons for withdrawing. Responses to this survey further informed our assessment of the program’s acceptability.

#### Efficacy

The following measures were administered at baseline, after course completion, and after the booster session to determine efficacy: Memory Strategy Knowledge [35], the Multifactorial Memory Questionnaire (MMQ) [36], Knowledge of Memory Aging Questionnaire [37], Depression Anxiety Stress Scale short form (DASS-21) [38], and contentment with ≤3 personal memory goals [16].

To further understand the intervention’s efficacy, as part of the postcourse evaluation, participants were asked, “What, if any, significant changes have you noticed in your life following the completion of the course?” The responses to this question were synthesized through thematic content analysis.

Finally, efficacy was also examined by reviewing participants’ responses on a discussion board during the booster session regarding how they were able to apply the knowledge and strategies taught in their everyday life, which strategies they use the most, in which situations they use these strategies, and which strategies they have not found useful.

### Statistical Analysis

All quantitative data analyses were conducted using SPSS Statistics (version 28, IBM Corp), apart from effect size calculations, which were performed in R (version 4.1.2, R Foundation for Statistical Computing) [39] using the *effectsize* package [40]. Unless otherwise stated, type I error (α) was set at the.05 level for all analyses. Exact *P* values are reported, apart from values <.001. The presence of univariate outliers was initially assessed using Tukey method, which defines outliers as values either larger than the 75th percentile plus 1.5 times the IQR or smaller than the 25th percentile minus 1.5 times the IQR. The application of this method suggested the presence of a small number of outliers (between 1 and 5 cases) on the Knowledge of Memory Aging Questionnaire, Memory Strategy Knowledge, MMQ, and goal satisfaction. The means and 5% trimmed means for these variables were compared and found to exhibit very little disparity. Thus, all cases were retained for the analyses presented here. Outliers were also detected in the DASS-21 at baseline (18/375, 4.8%), after the course (13/138, 9.4%), and after the booster (2/64, 3%). The deletion of these cases did not result in any meaningful changes to the model estimates; thus, all cases were retained for the following analyses.

To assess within-participant intervention efficacy at course completion and the maintenance of intervention effects after the booster session, linear mixed-effects (LME) analyses were performed for each outcome using the assessment time point (ie, baseline, after the course, and after the booster) as a fixed-effect predictor. Participants were treated as repeated effects to account for within-participant error term correlations. The baseline time point was set as the reference category in all the models. We used a first-order autoregressive covariance matrix and restricted maximum likelihood estimation in each model. In this approach, within-participant residual errors are correlated but are independent between participants. LME analysis was chosen over more traditional repeated measures methods, such as ANOVA, as LME allows for better handling of within-participant correlations across repeated measures and can better accommodate missing data [41,42]. As LME analyses use all available data for each participant, differences in group size across time points are less problematic compared with approaches such as ANOVA, which use list-wise deletion of cases. Mixed-effects models are also especially well suited to irregularly spaced repeated measures [41,43], which is the case in this study. All analyses were conducted using the maximum number of participants who had completed the respective measures at each time point; thus, the sample sizes for each analysis varied substantially over time. All analyses were rerun using a restricted data set containing only participants who completed the OPTIMiSE program (n=154). This resulted in no substantial changes to model estimates and did not affect the interpretation of results. Thus, all available data were retained for the final analyses presented here. Effect sizes (standardized mean differences in each outcome between baseline and each assessment time point) were calculated based on paired samples.

Independent content analysis was conducted by KP and CIM on participants’ written responses to “the most significant change in their life, if any, following OPTIMiSE.” The 2 researchers then discussed and reached a consensus on the major themes arising from the content analysis. These themes were confirmed through discussion with CB and MF. After this process, the themes were compared with those reported in 2 previous qualitative studies of face-to-face memory groups [19,44].

## Results

### Feasibility

In terms of recruitment, we contacted 181 U3A communities and 28 Probus clubs and sent emails to 10 people who were previously interested in LaTCH between September 11, 2020, and October 8, 2020 (28 days). Recruitment was rapid, with 123 people completing the web-based screening in the first 5 days and >350 people completing it within 2 weeks. Of the 633 individuals who completed the screening measures, 573 (90.5%) met the study’s inclusion criteria, of whom 387 (67.5%) provided informed consent and 357 (62.3%) completed all baseline questionnaires, which is twice our recruitment target of 153.

**Figure 1:**
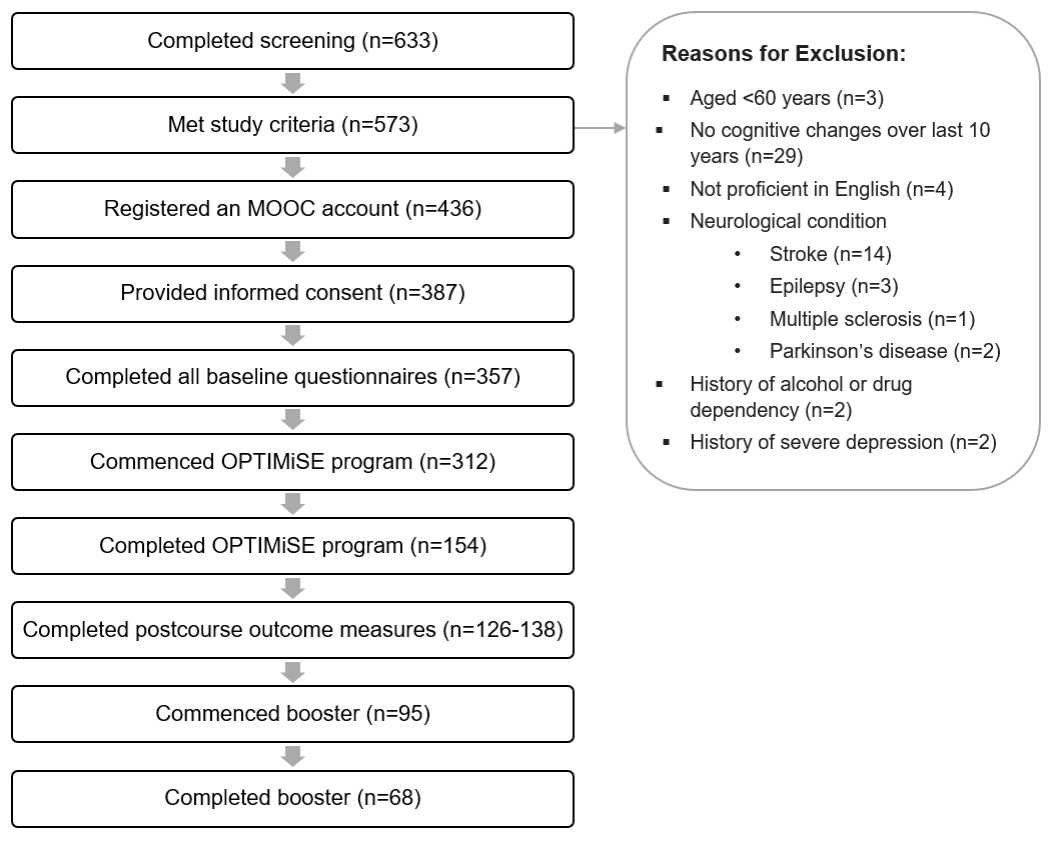
Participant flow during the study. MOOC: Massive Open Online Course; OPTIMiSE: Online Personalised Training in Memory Strategies for Everyday.

Figure 1 provides a detailed overview of participant flow throughout the study. In terms of attrition, of the 312 participants who commenced the OPTIMiSE program, 154 (49.4%) completed the program, and 68 (21.8%) completed the booster session. Attrition occurred in a gradual and consistent fashion across the duration of the program, with no specific dropout points.

Regarding the feasibility of data collection, missing data at any given time point were almost entirely due to attrition, rather than the noncompletion of measures by participants who were still actively taking part in the study. Across the postcourse and postbooster time points, outcome measures were completed by 88% of participants on average (range 81%-94%); refer to Multimedia Appendix 1 for further details. Feasibility is further supported because despite opportunities for feedback, no negative feedback was received regarding the appropriateness or burden of the assessments.

Feasibility was also informed by the amount of time taken to complete each module. The median time taken to complete each module was below the estimated 2 hours, apart from modules 2 and 3—module 1: 1 hour, 24 minutes (IQR 0 h, 53 min to 2 h, 41 min); module 2: 2 hours, 7 minutes (IQR 1 h, 17 min to 4 h, 23 min); module 3: 2 hours, 36 minutes (IQR 1 h, 26 min to 4 h, 27 min); module 4: 1 hour, 54 minutes (IQR 1 h, 13 min to 3 h, 8 min); module 5: 1 hour, 47 minutes (IQR 1 h, 8 min to 2 h, 54 min); module 6: 0 hour, 57 minutes (IQR 0 h, 29 min to 1 h, 44 min); and booster: 0 hour, 38 minutes (IQR 0 h,16 min to 1 h, 15 min)

In terms of ongoing cost feasibility, the main consideration is the staff time to support the delivery of OPTIMiSE. We had a dedicated email address for all inquiries, which received 388 emails between August 11, 2020, and March 8, 2021. Before course commencement, we received the following groups of emails: emails from organizations where we were recruiting (n=9), pre-enrollment inquiry emails (n=73), and baseline inquiry emails (from registered participants before course commencement; n=34). We also received 69 inquiries of people who missed out this time but were interested if the course was run again. During the course, we received 102 emails regarding technical issues, 7 emails with participant feedback, 30 withdrawal emails, 3 emails requesting results after the course, and 61 miscellaneous emails. To provide engagement with participants, time was also needed to monitor and respond to comments on the discussion boards and to provide a written weekly summary of the main themes that arose in comments on the previous module’s discussion boards. Finally, there was a panel discussion (featuring the core OPTIMiSE team members) as part of the conclusion module that was filmed in response to participants’ questions and provided reflection and general feedback about the course.

### Acceptability

In our a priori measure of acceptability—the question “Would participants recommend OPTIMiSE to others?”—we found very high levels of satisfaction, with 97.4% (150/154) of participants agreeing that they would recommend the course to others. Over 90% (145/154, 94.1%) of participants indicated that the program led to improved confidence in responding to memory challenges and that the program provided practical strategies that were helpful in everyday life (146/154, 94.8%). As demonstrated in Table 1, across all the overall evaluation items, participants generally reported positive experiences, with at least 83.1% (128/154) of participants agreeing with each item. Multimedia Appendix 2 also demonstrates that within each module, there was a high level of satisfaction with the content and delivery of the program.

When asked about the most useful aspects of the program, many participants indicated that these were the practical strategies, learning about how memory works, and the repetition and reinforcement of information (by presenting it in different modalities such as video, text, and quiz questions). Participants suggested that the discussion boards and animated LEGO videos were perhaps the least useful aspects, although opinions were divided on this. Several participants indicated that they did not find the acronyms to be useful, as they were overly complex. Similarly, participants reported that some of the optional materials that were provided were sometimes too detailed or too complex.

Suggestions for improvements to OPTIMiSE were gathered through the postcourse evaluation survey. The most common suggestion related to the amount of time that participants felt was necessary for the program each week, which many suggested was more than the recommended 2 hours, despite the objective median time to complete most modules being <2 hours. Alternatively, participants suggested that each module should be split across 2 weeks rather than 1; this was felt to be especially pertinent for the later modules, which were considered by many to be too long to complete within a 2-hour time frame. Many participants requested that written summaries be provided after every module, not just at the end of the course. Some participants suggested that the expert interviews could be shortened and revamped into a more casual style to prevent it from being overly long, being repetitious, or having an “academic” style.

Reasons for withdrawing from the program were primarily related to experiencing problems with technology, the program content not being applicable to the individual, or other external factors (eg, family commitments and health issues) impacting the ability to participate. Multimedia Appendix 3 contains some examples of the reasons for withdrawal.

To determine whether attrition was related to the baseline participant characteristics, we compared the characteristics of the participants remaining in the study at the following time points: baseline, course commencement, course completion, and booster completion. As presented in Table 2, there were no statistically significant differences across any of the baseline characteristics (age, gender, education, state of residence, birth country, primary language, web-based learning experience, family history of dementia, mood symptoms, general health, and general memory) across time points, with the exception of “experience using computers and the internet” (*P*=.02), in which a slightly lower proportion of booster completers reported moderate or higher level of experience using computers, compared with those who dropped out of the course at earlier time points.

**Table 1:** Online Personalised Training in Memory Strategies for Everyday (OPTIMiSE) overall evaluation items (n=154).

Items	Participants with strongly agree or agree responses, n (%)
I would recommend OPTIMiSE to others.	150 (97.4)
The OPTIMiSE online course was easily accessible.	153 (99.4)
The structure of OPTIMiSE was user-friendly.	151 (98.1)
The registration process was easy to follow.	153 (99.4)
Online learning gave me the flexibility to learn when it suited me.	154 (100)
The online learning material was of high quality.	151 (98.1)
The video presentations helped my learning.	128 (83.1)
The practical exercises were beneficial in applying learning to real life situations.	146 (94.8)
I understood what I was learning.	152 (98.7)
The optional learning materials provided useful and relevant information to supplement my learning.	127 (88.8^a^)
I feel less alone about my memory concerns.	146 (94.8)
The OPTIMiSE course improved my understanding of memory and how it changes across the lifespan.	152 (98.7)
The OPTIMiSE course can help individuals learn and apply memory strategies in everyday life.	152 (98.7)
I continue to apply my learning from the OPTIMiSE course in everyday life.	144 (93.5)
The OPTIMiSE course helped to normalise memory changes in older age.	148 (96.1)
The OPTIMiSE course improved my confidence in responding to memory challenges.	145 (94.1)

^a^n=143.

**Table 2:** Baseline characteristics of participants by stage completed.

Characteristics			Baseline (n=357)^a^		Commencers (n=312)^b^		Course (n=154)^c^		Booster (n=68)^d^		Comparison by completion status			*P* value	
Age (years), mean (SD)			72.14 (6.52)		72.13 (6.45)		71.68 (6.17)		71.88 (7.08).		*F*_4_=0.439			.73	
**Gender, n (%)^e^**												*χ*^2^_4_=3.1			.38
	Woman	280 (78.4)		244 (78.2)		126 (81.8)		57 (83.8)							
	Man	76 (21.3)		67 (21.5)		28 (18.2)		11 (16.2)							
**Education, n (%)**															
	Total tertiary	280 (78.4)		246 (78.9)		126 (81.9)		54 (79.4)		*χ*^2^_3_=14.6			.48		
**Region, n (%)^f^**												*χ*^2^_3_=1.9			.59
	Metropolitan	266 (74.5)		232 (74.3)		117 (76)		48 (70.6)							
	Regional	84 (23.5)		73 (23.4)		34 (22.1)		20 (29.4)							
**Country of birth, n (%)**															
	Australia	249 (69.7)		219 (70.2)		112 (72.7)		49 (72.1)		*χ*^2^_3_=12.6			.92		
**Language, n (%)^g^**															
	English	355 (99.4)		310 (99.4)		153 (99.4)		68 (100)		*χ*^2^_3_=1.3			.74		
**Computer and internet experience, n (%)**															
	≥Moderate	318 (89)		276 (88.5)		141 (91.6)		57 (83.8)		*χ*^2^_3_=9.5			.02		
**Web-based learning experience, n (%)**															
	≥Moderate	131 (36.7)		114 (36.6)		63 (40.9)		28 (41.2)		*χ*^2^_3_=4.5			.21		
**Family history, n (%)^h^**															
	Yes	155 (43.4)		133 (42.6)		67 (43.5)		24 (35.3)		*χ*^2^_3_=2.8			.43		
**General health, n (%)**															
	≥Average	342 (95.8)		298 (95.5)		147 (95.4)		66 (97.1)		*χ*^2^_3_=10.5			.31		
**Day to day memory, n (%)**															
	≥Average	307 (86)		269 (86.2)		131 (85)		57 (83.8)		χ^2^_3_=10.0			.62		

^a^Baseline: baseline completer.

^b^Commencer: course commencer.

^c^Course: course completer.

^d^Booster: booster completer.

^e^1 participant at baseline responded “prefer not to answer” to the question of gender.

^f^A small proportion of participants (<2%) did not provide their postcode; therefore, their region could not be determined.

^g^Language: language spoken at home.

^h^Family history: family history of dementia or memory problems.

### Efficacy

**Figure 2:**
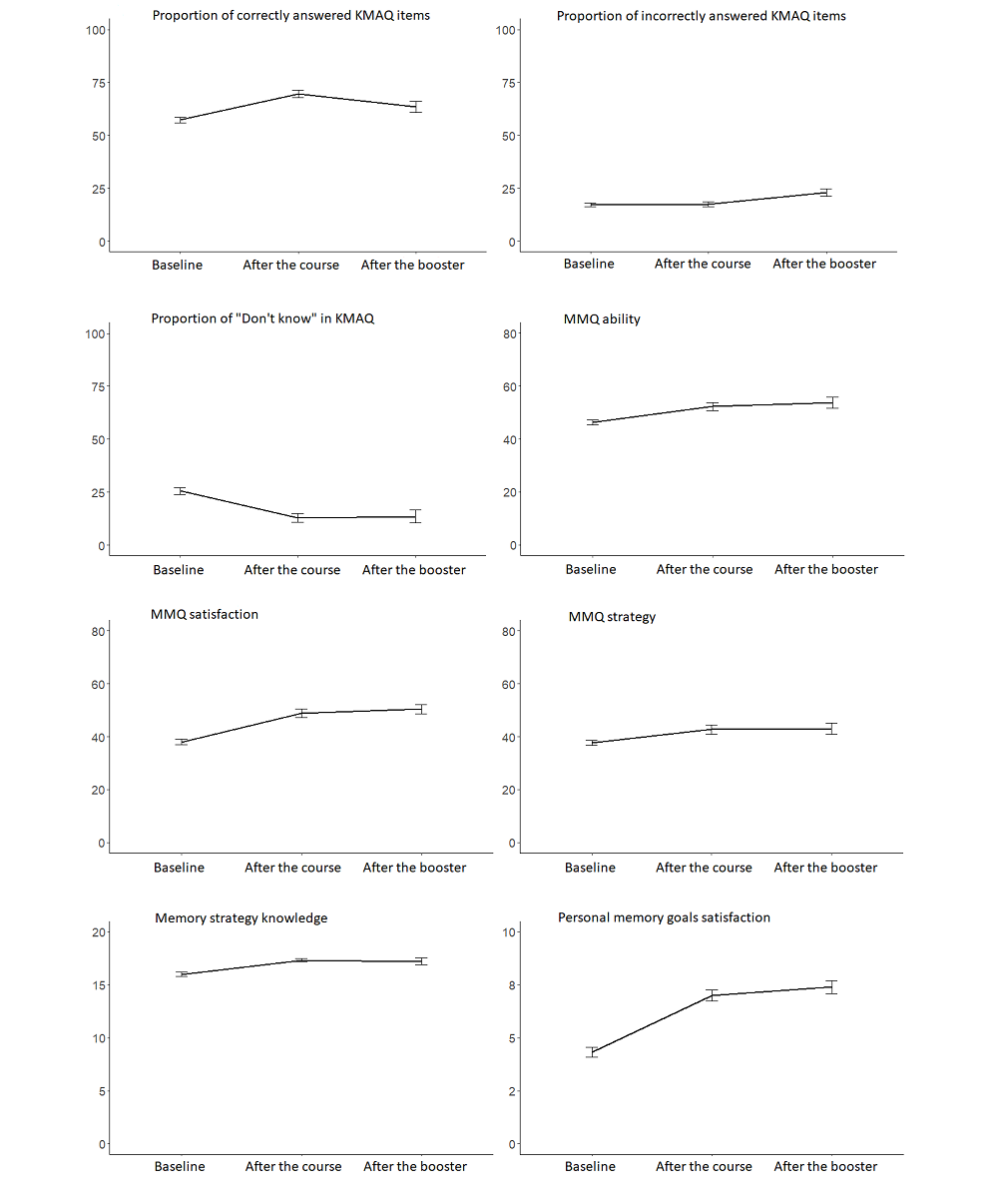
Efficacy of Online Personalised Training in Memory Strategies for Everyday (OPTIMiSE) demonstrated by changes in primary outcome measures after the course and after the booster. KMAQ: Knowledge of Memory Aging Questionnaire; MMQ: Multifactorial Memory Questionnaire.

As shown in Figure 2, the results of the LME analyses revealed that performance on all primary outcome measures improved after participation in the OPTIMiSE program (refer to Multimedia Appendices 1 and 4 for the detailed results). Satisfaction with personal memory goals improved significantly, with a large effect size, from an average of 4 (SD 1.78) out of 10 at baseline to 7 (SD 1.49) out of 10 at program completion (2-tailed t_235.45_=15.48; *P*<.001; Cohen *d*=1.24). This improvement was maintained at the 3-month follow-up (booster session) time point (average satisfaction=7, SD 1.19 out of 10; t_420.68_=13.15; *P*<.001; Cohen *d*=1.64). Similarly, performance on all 3 subscales of the MMQ improved significantly after the course (all with large effect sizes), and these improvements remained significant after the booster session. The MMQ ability mean score improved from 46.25 (SD 9.26) at baseline to 52.27 (SD 8.93) after the course (t_212.34_=9.78; *P*<.001; Cohen *d*=0.80) and 53.71 (SD 8.09) after the booster (t_306.76_=7.22; *P*<.001; Cohen *d*=0.83). MMQ satisfaction improved from 37.99 (SD 10.64) at baseline to 48.82 (SD 9.75) after the course (t_209.69_=16.12; *P*<.001; Cohen *d*=1.25) and 50.38 (SD 6.98) after the booster (t_313.17_=11.18; *P*<.001; Cohen *d*=1.29). MMQ strategy improved from 37.71 (SD 8.90) at baseline to 42.70 (SD 9.66) after the course (t_213.92_=9.60; *P*<.001; Cohen *d*=0.79) and 43.00 (SD 8.46) after the booster (t_302.87_=6.08; *P*<.001; Cohen *d*=0.90). Note that the size of the improvements on all the MMQ scales was similar to or larger than those in the previous face-to-face memory interventions [31].

Similarly, Memory Strategy Knowledge performance improved at both the postcourse and 3-month time points, although the degree of improvement was more modest than the improvement in other measures, increasing from a mean score of 16.00 (SD 2.00) at baseline to 17.34 (SD 0.96) after the course (t_191.37_=8.96; *P*<.001; Cohen *d*=0.67) and 17.22 (SD 1.20) after the booster (t_313.81_=5.73; *P*<.001; Cohen *d*=0.72). This moderate effect size contrasted with the large effect size observed in a previous face-to-face intervention [31]. The proportion of Knowledge of Memory Aging Questionnaire items answered correctly improved significantly from 57% (SD 13%) at baseline to 70% (SD 10%) after the course (t_199.99_=13.17; *P*<.001; Cohen *d*=0.96) and 64% (SD 10%) after the booster (t_291.19_=3.61; *P*<.001; Cohen *d*=0.26). The proportion of items answered incorrectly did not change after the course (17% vs 18%) but increased to 23% (SD 6%) at the booster time point (t_358.09_=5.75; *P*<.001; Cohen *d*=0.64), whereas the “don’t know” proportion decreased significantly from 26% (SD 15%) at baseline to 13% (SD 12%) after the course (t_197.85_=−11.48; *P*<.001; Cohen *d*=−0.85) and remained at this lower level after the booster (t_278.97_=−6.60; *P*<.001; Cohen *d*=−0.55).

The mean DASS-21 total score decreased (indicating improvement) significantly from 16.81 (SD 13.82) at baseline to 12.19 (SD 12.72) after the course (t_217.90_=−4.91; *P*<.001; Cohen *d*=−0.35). At the 3-month follow-up time point, the mean DASS-21 score of 14.06 (SD 14.54) was lower than that at baseline, although this reduction did not reach significance (t_294.55_=−1.95; *P*=.05; Cohen *d*=−0.07). Examining the DASS-21 subscale scores individually revealed that the mean depression subscale score was significantly lower after the course (5.72, SD 5.94 vs 3.83, SD 5.38; t_217.34_=−4.24; *P*<.001; Cohen *d*=−0.29); this reduction was maintained at the booster time point, although the size of the effect was small (3.84, SD 5.88; t_307.35_=−2.60; *P*=.01; Cohen *d*=−0.09), and the average was within the normal range at all time points. The anxiety subscale mean score also decreased significantly after the course (t_211.61_=−3.73; *P*<.001; Cohen *d*=−0.27), but this reduction was not maintained at the booster assessment time point (t_313.63_=−1.85; *P*=.07; Cohen *d*=−0.24). The same pattern of findings was observed for the stress subscale after the course (t_233.93_=−3.41; *P*<.001; Cohen *d*=−0.25) and after the booster (t_345.31_=−0.36; *P*=.72; Cohen *d*=0.10). The mean scores for both the anxiety and stress scales also fell within the normal range at all time points.

### Most Significant Change

Of the 154 participants, 131 (85.1%) provided a brief written response regarding the most significant changes in their lives after the completion of OPTIMiSE. The responses were examined using content analysis. A total of 8.4% (11/131) of participants reported no significant changes in their lives after the course. From the remaining 91.6% (120/131) of responses, several themes related to the course objectives as well as some unexpected themes were identified, as shown in Table 3.

Two themes were identified related to the course objective of “to learn and apply effective memory strategies in everyday life.” Many participants noted a change in their *strategy use* in everyday life, including using strategies explicitly taught in OPTIMiSE, such as paying attention, staying calm, organization, spaced retrieval, association, using external aids, circumlocution, reducing the amount of information to remember, and focusing on remembering the important things. The other theme related to this course objective was *improvements in daily life*, which included noting that OPTIMiSE had assisted in areas such as remembering names, remembering appointments, spending less time looking for lost objects, learning a new language, and gaining a better understanding of sleep. This theme is akin to the theme of *generalization* reported to arise after participation in the face-to-face memory groups [44].

The other course objective of OPTIMiSE was “to understand memory, how it works, and how it changes across the lifespan,” with an associated theme of *reduced concern about memory* arising from participant responses. Many participants noted that one of the most significant changes for them was worrying less about their memory; feeling more relaxed; and, particularly, being less concerned that any memory slips were a sign of underlying dementia. This theme relates to those of *acceptance and normalizing* [44] and *reduced anxiety* [19] reported in the face-to-face groups.

Two additional themes were identified in participants’ responses that were unrelated to the course objectives. The first of these was *confidence and self-efficacy*. Often, participants spoke about how reduced concern about memory led to greater confidence, frequently noting that they had skills and strategies they could use to manage any everyday memory lapses. There was a sense of trying to use memory strategies rather than giving up. For some, this also meant that they now felt enthusiastic about participating in other courses or felt more confident about coping with new technology. This relates to the themes of *coping and self-efficacy* [44] and *increased self-confidence and self-efficacy* [19] reported in the face-to-face interventions.

The final theme identified was *sharing and shame busting with others*. Participants reported that the course had led to them being able to better support their spouse’s memory, enabled them to joke with friends about memory issues, and empowered them to challenge the age stereotypes they encountered from family members. This theme has some overlaps with the theme of *improved relationships* reported in the face-to-face groups [19].

**Table 3:** Content analysis of the most significant changes after Online Personalised Training in Memory Strategies for Everyday (OPTIMiSE).

Objective and theme		Quote	Related themes: LaTCH^a^ studies
**Learn and apply effective memory strategies in everyday life**			
	Strategy use	“I have become more organised re glasses, keys and notice where I park my car. I do not run around in a panic. I no longer panic if I can’t remember a word or worry that I am losing it. I now substitute another word.”	N/A^b^
	Improvements in daily life	“I spend less time looking for lost objects.”	Generalization^c^
**Understand memory, how it works, and how it changes across the life span**			
	Reduced concern about memory	“I no longer have the fear that every memory glitch is an automatic indicator of dementia especially after realising that memory loss afflicts people almost half my age.”	Acceptance and normalizing^c^ Reduced anxiety^d^
**Themes unrelated to course objectives**			
	Confidence and self-efficacy	“Confidence, relaxed and determined to continue with the strategies I’ve learned.”	Coping and self-efficacy^c^ Increased self-confidence and self-efficacy^d^
	Sharing and shame busting with others	“More confident challenging ageist comments around memory.”	Improved relationships^d^

^a^LaTCH: La Trobe and Caulfield Hospital.

^b^N/A: not applicable.

^c^Matthews et al [44].

^d^Kinsella et al [19].

### Impact in Everyday Life

Responses reflecting the application of the course knowledge and strategies in everyday life were received from 63% (43/68) of participants who completed the booster session. All respondents indicated that participation in OPTIMiSE was beneficial and that the knowledge and strategies taught in the course were applicable to their everyday lives. Many participants reported reduced anxiety about memory concerns and an associated increase in self-confidence related to memory ability in everyday situations. Responses indicated that this reduced level of worry stemmed from an improved understanding about memory, how it works, and how it changes throughout life, in addition to knowing practical memory strategies that can be deployed in real-life situations. The specific strategies mentioned most often by participants as being useful included using external memory aids, staying calm and not worrying about memory issues when they occur, and actively concentrating when taking in information. The responses suggested that OPTIMiSE also served to reinforce participants’ existing memory strategies, specifically using external memory aids and keeping important objects in a specific place. Although the use of external memory aids was the most-nominated useful strategy, no particular strategy emerged as being the most (or least) useful for most participants. Several participants stated that not all strategies were especially relevant to their own personal needs. For instance, the use of acronyms was mentioned by several participants as not being particularly useful, whereas others found this strategy applicable and effective in more structured learning, such as learning a new language. More details related to the impact of OPTIMiSE on everyday life, including both successful and unsuccessful strategies, are presented in Multimedia Appendix 5. Of 43 participants, 2 (5%) participants noted that in some (unspecified) everyday situations, they were unable to apply memory strategies; some participants also noted some areas in which they were still unable to successfully apply memory strategies, including losing belongings (n=3, 7%), recalling the right word when needed (n=1, 2%), remembering information in the longer term (n=1, 2%), and remembering new information from a book (n=1, 2%).

## Discussion

### Principal Findings

OPTIMiSE—a 6-week web-based memory strategy training program—was shown to be feasible, acceptable, and efficacious for older adults with cognitive concerns. In terms of feasibility, a strong demand for the program was evident in the quick and relatively effortless recruitment. More than 600 people registered interest in OPTIMiSE over only 28 days, well exceeding our recruitment goal of 153, even though recruitment was only conducted through 2 organizations (U3A and Probus). Attrition was evenly distributed across the main course period, suggesting that there were no specific modules that were associated with greater attrition. Although the attrition rate is greater than that usually observed for in-person interventions [31], it is comparable with that observed for other web-based courses with set time frames [45,46]. A similar web-based memory intervention for older adults demonstrated less attrition [27]; however, timelines for completing the intervention were not fixed, suggesting that more flexibility around completion times may improve retention. Greater attrition was observed for the 3-month booster session, which only 21.8% (68/312) of the participants completed. The outcome measures for OPTIMiSE appeared feasible, with minimal missing data.

The program was acceptable to the target group, with 97.4% (150/154) of participants reporting that they would recommend it to someone else and 83.1% (128/154) of participants providing positive evaluations across all aspects of the program evaluated. This was similar to the end-of-course ratings in another web-based memory program for older adults [27]. We also asked participants to rate various aspects within each module, finding that the majority of evaluations were positive across all aspects and modules. Areas for improvement included lessening the amount of time needed to complete each module (even though the median time for the completion of most modules was below the expected 2-hour commitment), providing written summaries following each module, and shortening the length of some of the expert videos. Regarding reasons for withdrawal, although some participants withdrew owing to technical issues, most of the reasons were the same as those observed for in-person interventions (eg, change in personal circumstances and content not as expected).

OPTIMiSE was also efficacious, with postintervention improvements seen across all outcome measures, including knowledge of memory and aging, memory strategy knowledge and use, self-reported memory ability, achievement of personal memory goals, contentment with memory, and mood symptoms, and effect sizes similar to or larger than those observed after in-person interventions [31]. Content analysis of the most significant changes reported by participants after the intervention revealed themes consistent with the course objectives (*strategy use, improvements in daily life,* and *reduced concern about memory*), as well as the additional themes of *confidence and self-efficacy* and *sharing and shame busting with others*. These themes overlap substantially with those arising from previous in-person memory interventions using the most significant change analysis [19,44].

After OPTIMiSE, an additional theme of learning specific strategies was identified, whereas the theme of shared experiences, present after in-person interventions, was missing. This likely reflects the connections between individuals who are in the same room at the same time, compared with completing a course asynchronously with the only interaction via discussion boards. When developing OPTIMiSE, we aimed to balance increased access, cost-effectiveness, and resource effectiveness through web-based asynchronous delivery with ensuring that participants could communicate with peers, given the importance of peer comparison and interaction to foster the normalization of memory concerns [14,15]. In this study, despite shared experiences not being a commonly reported most significant change, normalization of memory concerns still appears to have occurred, with reduced worry and concern about memory and the possibility of dementia being a prominent theme. Participants also reported increased self-efficacy and confidence. Further research into the sense of connection among participants in asynchronous web-based interventions and how this relates to the effectiveness of the intervention, particularly around reducing concern and improving self-efficacy, is warranted.

The themes of reduced anxiety and increased confidence in memory were also seen in responses during the booster session regarding the application of knowledge and strategies from OPTIMiSE into everyday life. Many participants reported that they had been able to select strategies from OPTIMiSE that worked for them and applied these beneficially in their everyday lives. Some participants noted that although they had used the strategies throughout the course, they had not been applying them after the course ended. Most participants did not provide reasons for not maintaining the use of the strategies, although some mentioned being busy during the summer months between course completion and the booster session. Further exploration of potential barriers to maintaining the application of strategies, perhaps using in-depth qualitative interviews, would be helpful in understanding what does and does not facilitate the implementation of these strategies in everyday life.

There was no consensus regarding which strategies were the most or least useful. Increased confidence and paying attention or focusing on information were reported commonly, but all the strategies discussed in OPTIMiSE were mentioned by at least one participant, reflecting the diversity of needs even within this relatively homogenous cohort. Thus, teaching a wide range of different strategies is important, as different techniques will suit different individuals and different settings, as previously noted [47]. This is also consistent with previous meta-analytic reports that greater gains are made by teaching multiple memory strategies rather than focusing on single-strategy training [48].

### Limitations

This study has several limitations. First, it was not a randomized controlled trial but rather a pre-post pilot study. Hence, we did not have any control for potential natural changes in the outcome measures over time, although we would not expect the measures to improve without intervention. All questionnaires had high test-retest reliability, indicating stability over time [49]. We did not use any objective cognitive measures, which might have been expected to show practice effects. The lack of objective cognitive measures may be considered a limitation by some, but this was intentional, as OPTIMiSE was designed to assist participants in their memory in everyday life, not in test performance, and the link between improved test performance and improved performance in everyday life is tenuous at best [20]. Instead, we focused on self-reported knowledge, strategy use, contentment, and performance in everyday life as well as indicators of any significant changes after OPTIMiSE. The extension of these outcome measures to include the evaluation of strategy use in daily life, for example, using daily diary questions [47,50] or using ecological momentary assessment [47,51], would be a valuable future contribution. Ecological momentary assessment could be conducted via smartphone prompts several times per day, asking participants to log any memory strategy use, which strategies were used, and for what task the strategies were used.

Another limitation was the use of a convenience sample, which was highly educated, had high levels of computer literacy, and limited cultural diversity. An important next step will be to target recruitment more widely and examine the feasibility and acceptability for people with less education and computer literacy and more varied cultural backgrounds. The consideration of providing further support to engage older adults with less familiarity with technology [52] will be important to provide wider access.

In terms of strengths, the OPTIMiSE pilot did reach some rural residents, enabling participation by those who would not have been offered a face-to-face group. It also enabled scale of economy by delivering content to over 300 participants, rather than to 8 to 12 participants in a typical in-person group intervention. Moreover, much of the content would be used in future renditions of OPTIMiSE. In terms of sustainability, as delivered in this study, OPTIMiSE has some ongoing costs related to participant recruitment and support and the sections of the course that involve interaction (discussion boards, weekly summaries, and end-of-course panel discussion). Compared with a synchronous intervention, which would also require ongoing costs related to participant recruitment and support, as well as facilitator time, these costs are likely to be minimal.

Although attrition was similar to that observed in other web-based studies, it was high compared with that observed for in-person interventions. Attrition may be reduced by providing more information to participants before registration for the intervention (as many participants withdrew before giving consent) and providing accurate information regarding the duration of modules, given that the length required was a commonly suggested improvement. More flexibility around time to complete the course may also assist, given the lower attrition rate observed in another web-based memory study without a fixed time frame [27]. Nevertheless, relatively high levels of attrition may be par for the course for fixed-length web-based studies, suggesting that such studies need to account for this by recruiting and screening many more participants than will complete the course. Fortunately, if attrition is less than expected, there would be minimal (if any) impact on the resources required for an asynchronous web-based study.

Attrition impacts our primary measures of acceptability, which were administered at the end of the course and hence completed by only those who finished the course. Thus, we can only confidently state that the program was acceptable to the completers. Having data only from intervention completers is a common issue in determining the acceptability of an intervention. We tried to account for this by asking those who withdrew to complete a short web-based form providing the reasons for withdrawal, which provided us with some data; however, this form was only completed by 18.3% (24/131) of participants. We did gather information about acceptability along the way through feedback on each individual module (Multimedia Appendix 2), which suggested that most aspects of each module were acceptable to participants, even to those who withdrew later.

Participant feedback suggests that only minor tweaks are necessary for future revisions of OPTIMiSE. These include reducing the length of modules where possible, providing accurate information about the expected time commitment for each module (especially those with more content), providing handouts throughout the course, and revamping some of the expert interviews to be less technical. Further adaptations may be needed for different target groups, for example, older adults with greater memory difficulties (such as those with mild cognitive impairment) and those from more diverse cultural backgrounds. Conversely, OPTIMiSE may be most suited for older adults dwelling in the community, with subjective concerns but only minimal objective changes in their cognition. It may fit as an early or low-intensity intervention within a stepped or staged care model, akin to the models used for the treatment of anxiety and depression, which are especially useful for those living in rural communities [53,54]. It should be noted that OPTIMiSE is not intended to replace clinical care for individuals with dementia or substantial cognitive impairments. For people with more substantial cognitive issues, such as mild cognitive impairment or dementia, in-person or synchronous telehealth interventions may be required.

### Conclusions

This newly developed asynchronous web-based cognitive intervention program is a promising tool for assisting in providing older adults with greater access to cognitive interventions. Although considerably less expensive than face-to-face interventions, consideration of sustainable funding is one of the challenges in implementing this evidence-based intervention into practice. Some avenues to explore include community health services compared with outpatient services (such as memory clinics). It may also be important to investigate a user-pays model. OPTIMiSE provides an encouraging step toward meeting the challenge of the World Health Organization’s recommendation that cognitive interventions be provided to older adults to reduce the risk of cognitive decline and dementia [55].

## Appendix

Multimedia Appendix 1 Descriptive statistics of the outcome measures at each assessment time point.

Multimedia Appendix 2 Online Personalised Training in Memory Strategies for Everyday (OPTIMiSE) module evaluation: percentage of strongly agree or agree responses.

Multimedia Appendix 3 Reasons for withdrawing from Online Personalised Training in Memory Strategies for Everyday (OPTIMiSE).

Multimedia Appendix 4 Estimates of fixed effects on outcome measures.

Multimedia Appendix 5 Participant feedback regarding the application of strategies to everyday life.

## Abbreviations

CONSORT: Consolidated Standards of Reporting Trials
DASS-21: Depression Anxiety Stress Scale short form
LaTCH: La Trobe and Caulfield Hospital
LME: linear mixed-effects
MMQ: Multifactorial Memory Questionnaire
OPTIMiSE: Online Personalised Training in Memory Strategies for Everyday
U3A: University of the Third Age

## Data Availability

The data sets generated and analyzed during this study are not publicly available because of ethical requirements but are available from the first (corresponding) author on reasonable request.
