# Supplementary material for: Examination of the Feasibility, Acceptability, and Efficacy of the Online Personalised Training in Memory Strategies for Everyday Program for Older Adults: Single-Arm Pre-Post Trial
Source: J Med Internet Res. 2023 Apr 20;25:e41712. doi: 10.2196/41712 (PMC10160943; doi:10.2196/41712)
Supplement: Multimedia Appendix 1 [file jmir_v25i1e41712_app1.pdf]

Multimedia Appendix 1

Descriptive statistics of outcome measures at each assessment time point

Measure	Timepoint	*n*	*M*	*SD*
DASS-21[a] Total (x2)	Baseline	378	16.81	13.82
	Post-course	138	12.19	12.72
	Post-booster	64	14.06	14.54
DASS-21[a] Depression (x2)	Baseline	378	5.72	5.94
	Post-course	138	3.83	5.38
	Post-booster	64	3.84	5.88
DASS-21[a] Anxiety (x2)	Baseline	378	3.31	4.34
	Post-course	138	2.19	3.35
	Post-booster	64	2.47	4.15
DASS-21[a] Stress (x2)	Baseline	378	7.78	6.28
	Post-course	138	6.17	5.85
	Post-booster	64	7.75	7.03
KMAQ[b] – Correct	Baseline	357	0.57	0.13
	Post-course	127	0.70	0.10
	Post-booster	61	0.64	0.10
KMAQ[b] – Incorrect	Baseline	357	0.17	0.08
	Post-course	127	0.18	0.07
	Post-booster	61	0.23	0.06
KMAQ[b] – Don't Know	Baseline	357	0.26	0.15
	Post-course	127	0.13	0.12
	Post-booster	61	0.13	0.12
MSK[c]	Baseline	338	16.00	2.00
	Post-course	125	17.34	0.96
	Post-booster	63	17.22	1.20
MMQ[d] Ability	Baseline	365	46.25	9.26
	Post-course	128	52.27	8.93
	Post-booster	62	53.71	8.09
MMQ[d] Satisfaction	Baseline	366	37.99	10.64
	Post-course	128	48.82	9.75
	Post-booster	63	50.38	6.98
MMQ[d] Strategy	Baseline	364	37.71	8.90
	Post-course	127	42.70	9.66
	Post-booster	61	43.00	8.46
Personal Memory Goals	Baseline	241	4.32	1.78
	Post-course	126	7.00	1.49
	Post-booster	61	7.40	1.19

[a]DASS-21 (x2): Depression Anxiety Stress Scale 21-item short form (scores are doubled to allow comparison with the full DASS)

[b]KMAQ: Knowledge of Memory Aging Questionnaire

[c]MSK: Memory Strategy Knowledge

MMQ[d]: Multifactorial Memory Questionnaire
