# Supplementary material for: Examination of the Feasibility, Acceptability, and Efficacy of the Online Personalised Training in Memory Strategies for Everyday Program for Older Adults: Single-Arm Pre-Post Trial
Source: J Med Internet Res. 2023 Apr 20;25:e41712. doi: 10.2196/41712 (PMC10160943; doi:10.2196/41712)
Supplement: Multimedia Appendix 2 [file jmir_v25i1e41712_app2.pdf]

Multimedia Appendix 2

Online Personalised Training in Memory Strategies for Everyday (OPTIMiSE) module evaluation: percentage of strongly agree or agree responses.

	**Introduction**	**1**	**2**	**3**	**4**	**5**	**6**	**Booster**
	(n=227)	(n=218)	(n=187)	(n=188)	(n=166)	(n=162)	(n=174)	(n=68)
The module's objectives were well defined	n/a	98.1	99.5	97.9	99.4	99.4	99.4	95.6
The information and tasks enabled me to achieve the module's objectives	n/a	91.0	89.8	89.4	91.0	95.7	95.4	86.8
The material was easy to follow.	96.8	98.2	93.6	96.6	98.2	98.8	100	97.1
The amount of information was appropriate[a].	88.5	84.8	87.7	79.3	84.8	93.8	95.4	89.7
The information was appropriate in complexity.	n/a	90.3	87.6	85.6	90.3	94.4	96.6	89.7
The video presentations helped my learning.	n/a	74.5	84.4	76.6	74.5	84.5	n/a	79.4
The practical exercises were beneficial in applying my knowledge into practice.	n/a	84.2	89.8	86.7	84.2	88.2	n/a	n/a
The optional learning materials provided useful and relevant information to supplement my learning.	n/a	78.0	81.4	78.5	78.0	86.1	n/a	n/a
Overall, the module adds to the MOOC learning objectives[b].	90.3	97.6	98.4	97.3	97.6	99.4	97.7	86.8

Notes: n/a: not applicable; MOOC: massive open online course. [a]Percentages in this row indicate those who responded to the question with "about right". [b]The learning objectives were understanding about how memory works and how it changes across the lifespan, and knowledge of effective memory strategies and their application in everyday life. For the Introduction, this question relates to the overall understanding of what will be covered in OPTIMiSE modules.
