# Supplementary material for: Examination of the Feasibility, Acceptability, and Efficacy of the Online Personalised Training in Memory Strategies for Everyday Program for Older Adults: Single-Arm Pre-Post Trial
Source: J Med Internet Res. 2023 Apr 20;25:e41712. doi: 10.2196/41712 (PMC10160943; doi:10.2196/41712)
Supplement: Multimedia Appendix 3 [file jmir_v25i1e41712_app3.pdf]

Multimedia Appendix 3

Reasons for withdrawing from Online Personalised Training in Memory Strategies for Everyday (*OPTIMiSE).*

Theme	Quote
Learning new things	"I experienced problems in taking in the information and retaining it."
Technology	"My personal circumstances have changed and I don't have access to a computer. I find it very difficult to do an online course on a phone."
Time commitment	"There was more time involved than I anticipated."
	"I have just started learning a language and find I don't have the time for OPTIMiSE."
	"I simply did not have the time to participate fully, and felt that I was in a continuing catch-up mode."
	"It just took too long to complete modules"
Personal situation	"Unfortunately the timing for this course was not good for me as my [family member] passed away in the middle of it and has been very distracting. I enjoyed what little I managed to complete and would be happy to be added to your list for the next course. Thanks again for including me, I'm sure the course will improve most participants lives."
