# Supplementary material for: Examination of the Feasibility, Acceptability, and Efficacy of the Online Personalised Training in Memory Strategies for Everyday Program for Older Adults: Single-Arm Pre-Post Trial
Source: J Med Internet Res. 2023 Apr 20;25:e41712. doi: 10.2196/41712 (PMC10160943; doi:10.2196/41712)
Supplement: Multimedia Appendix 4 [file jmir_v25i1e41712_app4.pdf]

Multimedia Appendix 4

Estimates of fixed effects on outcome measures

Measure	Parameter	B	*SE*	*df*	*t*	*p*	95% CI	Cohen's *d* (95% CI)
DASS-21[a] Total (x2)	Intercept	16.81	0.71	428.38	23.58	< .001	15.41 – 18.21	
	Post-course	-3.87	0.79	217.90	-4.91	< .001	-5.43 – -2.32	-0.35 (-0.53, -0.18)
	Post-booster	-2.58	1.33	294.55	-1.95	.053	-5.19 – 0.03	-0.07 (-0.32, 0.17)
DASS-21[a] Depression (x2)	Intercept	5.72	0.30	433.88	18.81	< .001	5.12 – 6.32	
	Post-course	-1.52	0.36	217.34	-4.24	< .001	-2.22 – -0.81	-0.29 (-0.46, -0.12)
	Post-booster	-1.55	0.60	307.35	-2.60	.010	-2.72 – -0.38	-0.09 (-0.34, 0.15)
DASS-21[a] Anxiety (x2)	Intercept	3.31	0.22	434.88	15.29	< .001	2.89 – 3.74	
	Post-course	-1.00	0.27	211.61	-3.73	< .001	-1.53 – -0.47	-0.27 (-0.44, -0.10)
	Post-booster	-0.82	0.44	313.63	-1.85	.065	-1.68 – 0.05	-0.24 (-0.49, 0.01)
DASS-21[a] Stress (x2)	Intercept	7.78	0.33	455.27	23.93	< .001	7.14 – 8.42	
	Post-course	-1.41	0.41	233.93	-3.41	< .001	-2.23 – -0.6	-0.25 (-0.42, -0.08)
	Post-booster	-0.24	0.68	345.31	-0.36	.719	-1.58 – 1.09	0.10 (-0.14, 0.35)
KMAQ[b] – Correct	Intercept	0.57	0.01	410.79	89.92	< .001	0.56 – 0.59	
	Post-course	0.10	0.01	199.99	13.17	< .001	0.09 – 0.12	0.96 (0.75, 1.18)
	Post-booster	0.05	0.01	291.19	3.61	< .001	0.02 – 0.07	0.26 (0.01, 0.52)
KMAQ[b] – Incorrect	Intercept	0.17	0.00	441.66	40.82	< .001	0.16 – 0.18	

Measure	Parameter	B	*SE*	*df*	*t*	*p*	95% CI	Cohen's *d* (95% CI)
	Post-course	0.00	0.01	204.19	0.40	.692	-0.01 – 0.01	0.03 (-0.14, 0.21)
	Post-booster	0.06	0.01	358.09	5.75	< .001	0.04 – 0.07	0.64 (0.37, 0.93)
KMAQ[b] – Don't Know	Intercept	0.26	0.01	404.65	33.04	< .001	0.24 – 0.27	
	Post-course	-0.10	0.01	197.85	-11.48	< .001	-0.12 – -0.09	-0.85 (-1.05, -0.64)
	Post-booster	-0.10	0.01	278.97	-6.60	< .001	-0.13 – -0.07	-0.55 (-0.83, -0.28)
MSK[c]	Intercept	16.01	0.10	421.03	167.06	< .001	15.82 – 16.19	
	Post-course	1.21	0.13	191.37	8.96	< .001	0.94 – 1.47	0.67 (0.47, 0.88)
	Post-booster	1.18	0.21	313.81	5.73	< .001	0.78 – 1.59	0.72 (0.43, 1.01)
MMQ[d] Ability	Intercept	46.25	0.48	427.23	96.38	< .001	45.3 – 47.19	
	Post-course	5.79	0.59	212.34	9.78	< .001	4.62 – 6.96	0.80 (0.60, 1.01)
	Post-booster	6.95	0.96	306.76	7.22	< .001	5.05 – 8.84	0.83 (0.54, 1.12)
MMQ[d] Satisfaction	Intercept	37.99	0.54	429.27	70.94	< .001	3.89 – 7.61	
	Post-course	11.00	0.68	209.69	16.12	< .001	3.89 – 7.61	1.25 (1.02, 1.49)
	Post-booster	12.25	1.10	313.17	11.18	< .001	3.89 – 7.61	1.29 (0.96, 1.64)
MMQ[d] Strategy	Intercept	37.71	0.48	426.37	79.25	< .001	36.78 – 38.65	
	Post-course	5.54	0.58	213.92	9.60	< .001	4.4 – 6.67	0.79 (0.60, 1.00)
	Post-booster	5.75	0.95	302.87	6.08	< .001	3.89 – 7.61	0.90 (0.60, 1.21)
Personal Memory Goals	Intercept	4.32	0.10	422.54	41.24	< .001	4.12 – 4.53	

Measure	Parameter	B	*SE*	*df*	*t*	*p*	95% CI	Cohen's *d* (95% CI)
	Post-course	2.69	0.17	235.45	15.48	< .001	2.35 – 3.03	1.24 (0.99, 1.50)
	Post-booster	3.06	0.23	420.68	13.15	< .001	2.6 – 3.51	1.64 (1.24, 2.07)

[a]DASS-21 (x2): Depression Anxiety Stress Scale 21-item short form (scores have been doubled to allow comparison with the full DASS)

[b]KMAQ: Knowledge of Memory Ageing Questionnaire

[c]MSK: Memory Strategy Knowledge

[d]MMQ: Multifactorial Memory Questionnaire
