# Supplementary material for: Examination of the Feasibility, Acceptability, and Efficacy of the Online Personalised Training in Memory Strategies for Everyday Program for Older Adults: Single-Arm Pre-Post Trial
Source: J Med Internet Res. 2023 Apr 20;25:e41712. doi: 10.2196/41712 (PMC10160943; doi:10.2196/41712)
Supplement: Multimedia Appendix 5 [file jmir_v25i1e41712_app5.pdf]

Multimedia Appendix 5

Participant feedback regarding application of strategies to everyday life.

Successful Strategies	Situation/s	*n*
External memory aids	Electronic calendars (computer or phone) to provide scheduled reminders of upcoming appointments.<br>Cooking timers.<br>Paper diaries or calendars for scheduling appointments.<br>Paper diaries for recording details of events and conversations.<br>Written to-do lists, shopping lists, etc.<br>Written notes reminding of tasks to complete or upcoming appointments.	20
Concentration / mindfulness	In conversation with others.<br>When learning new information such as people's names.<br>Remembering surroundings such as when parking the car.	19
Staying calm	When experiencing a memory glitch, such as trying to recall a specific word or someone's name.<br>Understanding that memory glitches are normal and understanding why they occur.<br>Understanding that my experiences are shared by others.	19
Spaced retrieval	Phone numbers.<br>Peoples' names.<br>Learning new information, such as a language, etc.	13
Association	When meeting new people, to try and remember their names.	10
Specific place for important items	Keys (house, car, etc.) – specific location near the front door.<br>Reading glasses – specific location such as kitchen bench, etc.<br>Notes, lists, reminders, etc. – central place such as the fridge.	10
Repetition	When meeting new people, repeating their names.<br>Memorising lists, e.g., shopping lists.	9
Prioritise	Identifying and focusing on important information, disregarding less important information.	8
Acronyms	To help establish set routines, such as when leaving/locking up the house, going to bed, etc.	6

	In everyday situations involving lots of things to remember.	
Organisation	Filing important paperwork.<br>Labelling items.<br>Creating weekly plans and to-do-lists.	3
PQRST	Learning a new language.<br>Taking in large amounts of new information.	3
Verbalise	Remembering the location of objects, e.g. "I am putting this scarf on the back of the chair."<br>Intended actions, e.g. "I am going to the kitchen to get X."	3
Alphabet	In conversation with others, mentally go through the alphabet to find the right word or name.	2
Active listening	In conversation with others.	1
Ask others	In conversation with others, ask when I don't remember information, e.g., names or events.	1
Chunking	*None specified**	1
Circumlocution	Trying to recall a specific word, e.g., when in conversation with others.	1
Implementation intentions	*None specified**	1
SMART goals	Communicating with friends and family.	1
Unsuccessful Strategies	Situation/s	*n*
Acronyms	Acronyms seen as an extra thing to remember, adding to memory load.	4
Association	Can be difficult to create an effective associative relationship.	3
Specific place for important items	Busy situations with lots of distractions.	2
Implementation intentions	*None specified**	1

* The strategies "Chunking" and "Implementation intentions" were identified as useful, however the situations in which these strategies were useful were not specified by participants.
